# Feasibility and sustainability of a nurse-led intervention to integrate HPV vaccination into medical processing for active-duty Soldiers

**DOI:** 10.1080/21645515.2022.2153536

**Published:** 2022-12-20

**Authors:** Emily Penick, Brigid K. Grabert, Emma Stockton, Hannah Prentice-Dunn, Marion Ward, Trinita Kirk, Melissa B. Gilkey

**Affiliations:** a Department of Obstetrics & Gynecology, Womack Army Medical Center, Fort Bragg, NC, USA; b Lineberger Comprehensive Cancer Center, University of North Carolina School of Medicine, Chapel Hill, NC, USA; c Department of Health Behaviors, University of North Carolina at Chapel Hill, Chapel Hill, NC, USA; d Medical One Stop, Womack Army Medical Center, Fort Bragg, NC, USA

**Keywords:** Health communication, human papillomavirus infections/prevention and control, human papillomavirus vaccine, quality of health care

## Abstract

To increase Soldiers’ access to HPV vaccination, we evaluated the feasibility and sustainability of a nurse-led intervention to integrate HPV vaccination into medical processing procedures for Soldiers. We partnered with nursing staff to introduce HPV vaccine into existing vaccination services at a nurse-led clinic that serves Soldiers at Fort Bragg, North Carolina. In addition to stocking the vaccine, the intervention included training nursing staff (*n* = 11) strategies for recommending HPV vaccination for Soldiers ages 18–26. We conducted surveys of nursing staff to assess their perspectives on feasibility. Nursing staff tracked HPV vaccine uptake among Soldiers for 4 weeks post-training to assess adoption and again for 2 weeks at 4-month follow-up to assess sustainability. We assessed delivery cost as the cost of personnel time, vaccine doses, and other materials during the initial 4-week intervention period. Nursing staff agreed that recommending HPV vaccination fit in with medical processing procedures (mean = 4.6 of 5.0). Of the 516 Soldiers offered HPV vaccine in the 4 weeks following the training, 198 (38%) accepted and received the vaccine. Soldier ages 18–20 more often accepted HPV vaccination than older Soldier ages 21–26 (46% versus 32%, *p* < .01). Vaccine uptake was similar at follow-up, with 98 of 230 eligible Soldiers (43%) receiving HPV vaccine. The total delivery cost was $12,737, with an average cost per vaccine dose delivered of $64. Our findings suggest that training nursing staff to recommend and administer HPV vaccinations to Soldiers is feasible and warrants wider-scale testing as a strategy to protect soldiers from HPV-attributable cancers.

## Plain Language Summary

HPV vaccination is not required for US military service, and Soldiers’ uptake is low. We trained nursing staff at a large military clinic to recommend HPV vaccine to Soldiers using a nursing education intervention to integrate HPV vaccination into routine care for active duty Soldiers. Our findings suggest that training nursing staff to recommend and administer HPV vaccines to Soldiers is feasible and low cost, and may warrant wider-scale testing as a strategy for increasing military readiness and protecting Soldiers from HPV-attributable cancers. Until guideline and policy changes are implemented and HPV vaccine is required for military service, use of education strategies is one path to increasing HPV vaccine coverage among Soldiers to ensure protection from HPV-related diseases.

## Introduction

Improving human papillomavirus (HPV) vaccination coverage among active-duty Soldiers is an important goal for the US military. Soldiers, who are a relatively young population, are at elevated risk for HPV, given that this highly prevalent infection is typically acquired in late adolescence and early adulthood.^1^ HPV infections can progress undetected, eventually leading to cancers of the cervix, vagina, vulva, penis, anus, and throat.^2^ Even when treated successfully, HPV disease can take a personal and professional toll on Soldiers, since they must devote time to appointments and procedures and cannot be deployed during follow-up.^3,4^ HPV vaccination is a safe and effective way to prevent HPV cancers.^5^ Although vaccination during the preteen years is best, adolescents and young adults can still derive substantial benefits.^5–8^ For example, HPV vaccination at ages 17–30 reduces the risk of cervical cancer by about 50%.^8^

Despite the benefits, rates of HPV vaccine series initiation and completion remain low in the US, including among active-duty Soldiers.^9^ Although data is sparse, existing studies suggest that only about one-quarter (26%) of active-duty women, who were eligible for HPV vaccination in 2007–2017 initiated the series, despite having coverage for this benefit through military-sponsored insurance plans.^10,11^ In contrast to vaccines for most other vaccine-preventable diseases, HPV vaccination is not required for military service.^11^ For this reason, Soldiers may lack convenient opportunities and provider recommendations to get HPV vaccine.

To address this gap, we designed and evaluated the feasibility and sustainability of a nurse-led intervention to introduce HPV vaccination into routine care for active-duty Soldiers visiting a large Army base medical processing center. Prior to this intervention, HPV vaccines were not stocked or administered at this location. Our aims were to assess our intervention’s impact on HPV vaccine uptake, as well as implementation outcomes such as acceptability and delivery cost. By providing novel data on a system-level intervention to improve HPV vaccine uptake, this research seeks to lay the groundwork for larger-scale efforts to improve Soldiers’ access to HPV vaccination and maximize their protection from HPV cancer.

## Methods

### Study setting and participants

Our study took place at the Medical One Stop clinic in Fort Bragg, North Carolina, a US Army installation. The Medical One Stop conducts all medical in- and out-processing at Fort Bragg, and provides care for approximately 23,000 Soldiers each year. This clinic is led by nursing staff with a Head Nurse (RN) overseeing daily functions. Clinic staffing is made up of RNs, LPNs, Physician Assistants, and Medical Support Assistants. The nursing staff team at the clinic ensure Soldiers meet assignment eligibility, including vaccination requirements. If gaps are identified, Medical One Stop nurses are trained to administer vaccinations at the time of initial contact, with a focus on vaccines required for military service. The Department of Defense administers 17 different vaccines to service members. Required vaccines include Adenovirus, Hepatitis A and B, Influenza, Measles, Mumps, and Rubella (MMR), Meningococcal, Poliovirus, Tetanus-Diphtheria, and Varicella.^12^ Additional vaccines such as Anthrax, Japanese encephalitis, and Smallpox are given based on risk and occupation.^12^ Prior to our intervention, the Medical One Stop did not stock the HPV vaccine.

### Intervention

#### Nursing staff training

In March 2021, one author (EP), a Ft. Bragg physician, assisted by one Ft. Bragg physician resident, delivered a one-hour training about HPV vaccine communication and administration to nursing staff (*n* = 11) of the Medical One Stop. All nursing staff working in the Medical One stop were eligible to participate in the training. The training outlined the importance of eligible Soldiers receiving HPV vaccine, how to administer and document HPV vaccination, and how to recommend HPV vaccination and effectively address Soldiers’ questions and concerns. The training adapted content from two evidence-based interventions, the Announcement Approach Training and DOSE HPV.^13–15^ These interventions instruct clinical staff to use presumptive recommendations, presenting HPV vaccine as the default choice in routine health care, along with other vaccines for which the patient may be due. Due to ethical considerations, we modified this approach to inform soldiers that HPV vaccination is not mandatory per Department of Defense orders, but is strongly recommended. After announcing that the Soldier was due for HPV vaccination, nursing staff were instructed to connect with and counsel Soldiers if they had concerns about receiving the vaccine.^13^ We encouraged nursing staff to use research-tested messages,^15–17^ focusing on the vaccine’s safety and efficacy, as well as cancer prevention.

The training was interactive and included role-play and group discussion. Role-play interaction allowed nursing staff to practice the use of the presumptive HPV vaccine statement “You’re due for [#] vaccines today that protect against [X, X, X] and HPV cancers. [X, X, and X] vaccines are required for service, and we also strongly recommend the HPV vaccine. We’ll get you caught up on these vaccines today. Any questions?” Staff also practiced answering common questions about HPV vaccination and how to navigate conversations regarding vaccine declination. Role-play occurred in pairs, with one participant playing the role of Soldier and the other of the nursing staff counselor. Interactive group discussions took place throughout the presentation, with time at the end of the training for a Question & Answer session. We provided nursing staff with handouts and reminder cards to aid in Soldier education regarding HPV vaccine and to assist with reminders for completion of the vaccine series.

#### Clinic procedures

In addition to training, the study team worked with nursing staff to stock HPV vaccines and integrate HPV vaccination into the clinic's flow. First, we developed a decision tree to identify Soldiers eligible for HPV vaccination as those who were ages 18–26 without a record of completing the HPV vaccine series, as documented by medical records or Soldiers’ self-report (see Supplemental Figure S1). Second, we worked with the Ft. Bragg pharmacy to order and stock the HPV vaccine at the Medical One Stop. Finally, we prepared nursing handouts on answering questions, as well as handouts and reminder cards for Soldiers.

### Data collection procedures and measures

#### Nursing staff perceptions

Nursing staff completed two brief online surveys, one immediately before and one immediately after the training. They reported their perceptions related to HPV vaccine delivery for Soldiers generally and our intervention specifically. Administered via Qualtrics (Qualtrics XM, Provo, UT), the surveys included statements that respondents rated using a 5-point response scale (1 = “strongly disagree” to 5 = “strongly agree”). We developed measures to assess the following domains:

##### HPV vaccine perceptions

Four statements on the pre- and post-training surveys assessed nursing staff perceptions related to HPV vaccine delivery for Soldiers. These measures assessed perceived importance of recommending HPV vaccine to Soldiers, perceived importance that Soldiers place on HPV vaccination, confidence in addressing Soldiers’ concerns about HPV vaccine, and intentions to routinely recommend HPV vaccine to Soldiers.

##### Intervention perceptions

Four statements in the post-training survey assessed nursing staff perceptions related to the acceptability of and intention to deliver the intervention. More specifically, these measures assessed the extent to which respondents agreed that presumptive recommendations fit into Soldier's in- and out-processing visits, that presumptive recommendations fit into a clinic workflow, that respondents planned to use presumptive recommendations, and that they would recommend the training to a colleague.

#### HPV vaccine delivery

We collected data on HPV vaccine delivery at two times: a 4-week period immediately following the training (April 1–30, 2021) and a 2-week period at 4-month follow-up (August 16–31, 2021). During these time periods, nursing staff collected data during patient visits using a tracking log. The clinic health nurse oversaw counseling and delivery of HPV vaccinations. Based on a combination of the medical record and Soldiers’ self-reports, nursing staff recorded each Soldier’s age and HPV vaccination status in terms of number of doses received previously. For Soldiers eligible for one or more additional doses of HPV vaccine, staff also recorded whether the Soldier accepted and received HPV vaccine. During the follow-up period alone, staff recorded Soldiers’ reasons for declining HPV vaccination, using a pre-specified list. Responses were as follows: wanting to learn more about the vaccine before vaccination, belief that the vaccine is not needed or necessary, concerns about receiving too many vaccines, concerns about safety/side effects, the Soldier was not sexually active, and other reasons.

We used tracking log data to derive:

##### Adoption

To assess the adoption of the intervention, we operationalized the measure as the proportion of eligible Soldiers who were offered and received any dose of HPV vaccine (first, second, or third) during the initial 4-week data collection period.

##### Sustainability

To understand the ability of nursing staff to continue to deliver HPV vaccine after the initial intervention period, we measured sustainability as the proportion of eligible soldiers who were offered and received any dose of HPV vaccine at 4-month follow-up.

#### Delivery cost

We assessed *delivery cost* from the perspective of the interventionists (here, the US Department of Defense) as the average cost per vaccine dose delivered during the initial 4-week intervention period. We determined the cost for staff time spent attending the training and other time spent by study staff preparing for the training, using individual staff salaries to determine an hourly rate and rounded to the nearest half hour. We calculated hourly rates by dividing annual salaries for each attendee and presenter by 2080, or 40 h a week for 52 weeks. For example, an RN with an annual salary of $95,000 had an hourly rate of $45.67. We included the cost of all HPV vaccine doses administered during the intervention period. Additionally, we included the cost of materials used in the training, including the handouts and reminder cards given to nursing staff. We did not include costs related solely to evaluation activities, such as the time needed to complete nursing staff surveys. We also did not include the staff time needed to deliver the HPV vaccine because staff were able to incorporate vaccination into their usual clinic routine without the need for additional hours. As the Medical One Stop clinic already stocked other vaccines, we did not include costs associated with refrigeration or storage, and no additional edits were required to the electronic health records.

The Womack Army Medical Center Human Research Protections Program Office reviewed this project for applicability of human subjects protections regulations and classified it as a process improvement project.

### Analyses

To evaluate *nursing staff perceptions*, we calculated the mean response scores for each survey item before and after training. For questions that were asked in both surveys, we compared responses using Wilcoxon-Mann-Whitney tests for significance to determine if nursing staff answers changed after the training. Statistical tests were two-tailed with a critical alpha of 0.05.

To evaluate *adoption* and *sustainability*, we calculated the proportion of Soldiers who received a dose of HPV vaccine of the total eligible Soldiers who were offered HPV vaccine. For the initial four-week data collection period, we used bivariate logistic regression to assess correlates (sex, age, and number of other vaccine doses delivered) of HPV vaccine uptake.

To evaluate the *delivery cost*, we calculated the overall cost of the intervention as the total cost of each intervention component (training preparation, intervention delivery, training materials, nursing staff materials, and HPV vaccine cost). We calculated the average cost per HPV vaccine dose as the total cost divided by the total number of doses delivered during the initial 4-week intervention period.

## Results

Eleven nursing staff of the Medical One Stop clinic completed the one-hour training on HPV vaccine recommendation and administration. Ten nursing staff completed the before-training survey, and 11 completed the after-training survey.

### HPV vaccine perceptions

**Figure 1.**
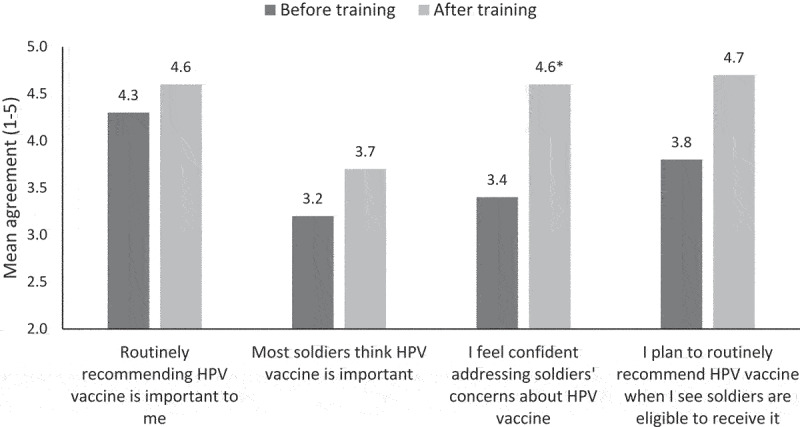
Nursing staff perceptions before and after training.*p<.05.^^^Included nurses, medical support assistants, and health technicians.

Mean scores on HPV vaccine perceptions ranged from medium to high agreement (Figure 1). Nursing staff's confidence about addressing Soldiers’ HPV vaccine concerns was higher after the training (before = 3.4 to after = 4.5, *p* < .05). Mean scores on other HPV vaccine perception measures trended higher after training compared to before training, but the increases were not statistically significant.

### Intervention perceptions

Nursing staff strongly agreed that the presumptive recommendation approach fit with in- and out-processing procedure visits (mean = 4.6) and fit into their clinic’s workflow (mean = 4.5). All nursing staff somewhat or strongly agreed that they planned to adopt the presumptive recommendation approach (mean = 4.7) and would recommend the training to their colleagues (mean = 4.6).

### HPV vaccine delivery

#### Adoption

**Figure 2.**
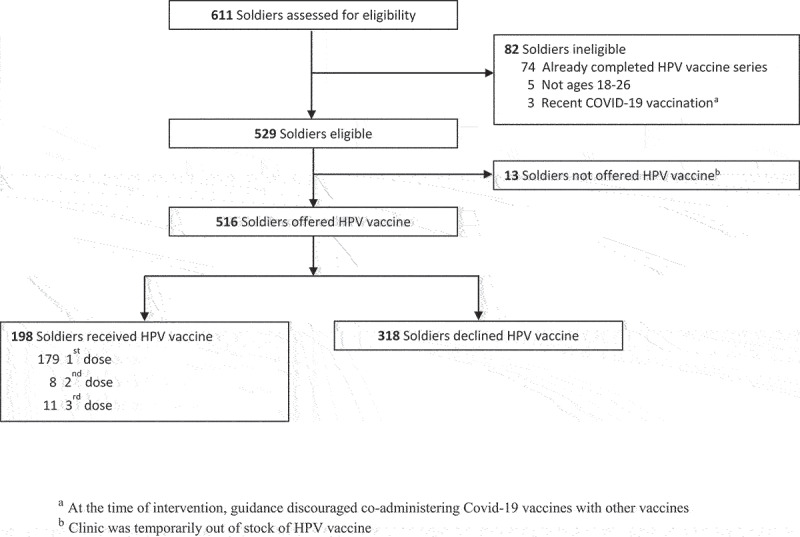
Flow diagram of soldiers assessed at Medical One Stop during 4-week intervention period.

A total of 611 soldiers presented for medical processing during the four-week intervention period (Figure 2). Of these, 84% (*n* = 516) were eligible to receive and were offered the HPV vaccine, while 13% (*n* = 82) were ineligible and 2% (*n* = 13) were not offered the HPV vaccine because of insufficient stock. Reasons for ineligibility were that soldiers had already completed the HPV vaccine series (*n* = 74), were not ages 18–26 (*n* = 5), or had recently received a Covid-19 vaccine (*n* = 3), which at the time was not recommended with other vaccines.

Of the 516 soldiers who were offered the HPV vaccine, 38% (*n* = 198) accepted and received the vaccine, including 179 first doses, 8 second doses, and 11 third doses (Table 1). Younger soldiers, ages 18–20, more often accepted HPV vaccine compared with those ages 21–26 years (46% versus 32%, *p* < .01). Neither sex nor the number of other vaccines given during the visit was associated with uptake.

**Table 1.** Characteristics of eligible Soldiers and associations with HPV vaccine receipt (*n* = 516).

					Bivariate association	
	*n* (%)		No. who received HPV vaccine/total in category (%)		Effect	(Std. error)
Sex^a^						
Male	427	(83)	160/427	(37)	Reference	
Female	88	(17)	38/88	(43)	0.06	(0.06)
Age (years)^b^						
18–20	226	(44)	104/226	(46)	Reference	
21–26	284	(56)	92/284	(32)	−0.14**	(0.043)
Number of other vaccines received at visit						
0	229	(44)	87/229	(38)	Reference	
1	183	(35)	70/183	(38)	0.00	(0.05)
2–3	42	(8)	18/42	(43)	0.05	(0.08)
Missing	62	(12)	23/62	(37)	–	–

Effect and adjusted effects are average marginal effects. Std. errors = robust Delta-method standard errors. Dashes (--) indicate not included in bivariate model.

*p < .05; **p < .01.

^a^Data missing for 1 soldier.

^b^Data missing for 6 soldiers.

#### Sustainability

During the two-week follow-up period, 256 Soldiers presented for medical processing, and 90% (*n* = 230) were eligible to receive and were offered HPV vaccine. Of these 230 eligible Soldiers, 43% (*n* = 98) accepted HPV vaccine, including 85 first doses, 8 second doses, and 5 third doses. When asked about their reason for HPV vaccine refusal, 45% of declining Soldiers (*n* = 59/132) indicated “other reason,” followed by “wanting to learn more about vaccine first” (19%) and “the vaccine is not needed/necessary” (16%, Table 2).

**Table 2.** Reasons for HPV vaccine declination among eligible soldiers who declined vaccination in a follow-up sample (*n* = 132)^a^.

	*n* (%)	
Want to learn more about it first	25	(19)
Not needed/necessary	21	(16)
Concern about getting too many vaccines at one time	11	(8)
Other concern about safety/side effects	8	(6)
Not sexually active	7	(5)
Other reason	59^a^	(45)

^a^1 soldier did not provide a reason.

### Delivery cost

The total cost of the initial 4-week intervention period was $12,737, with an average cost per vaccine dose delivered of $64 (Table 3). Personnel costs were $670, or 5% of the total cost, including 2 physician presenters and 11 Medical One Stop nursing staff attendees. Materials cost $86 (1%) and included vaccine reminder cards for soldiers, informational flyers about HPV vaccine for adults, and handouts and vaccination decision tree cards for nursing staff. The vaccine doses cost $11,981 (198 doses at $60.51/dose) and made up 94% of the total cost of the intervention.

**Table 3.** Delivery cost for HPV vaccine doses delivered during 4-week intervention period.

	Cost^a^
Total cost	$12,737
Materials^b^	$86
Personnel	$670
Presenters (*n* = 2)	$209
Attendees (*n* = 11)^c^	$461
HPV vaccine doses	$11,981 ($61/dose for 198 doses)
Cost per vaccine dose delivered	$64 ($12,737/198 doses)

^a^Costs rounded to the nearest US dollar.

^b^Includes handouts, flyers, nursing staff flow chart cards, soldier reminder cards.

^c^Includes one participant with unknown clinic staff role in which we imputed the mean salary of attendees ($45.02/hour).

## Discussion

The findings of our evaluation suggest that using a light-touch nursing education program is a highly feasible way to increase Soldiers’ access to HPV vaccination. During the initial intervention period, we found that a high proportion of Soldiers were eligible for HPV vaccination, and over one-third accepted a dose. Nursing staff reported that HPV vaccine recommendations and counseling fit easily into the clinic workflow, suggesting that our intervention was highly acceptable to staff. Furthermore, our training increased staff’s confidence in addressing Soldiers’ concerns about HPV vaccination. Importantly, we found that nursing staff continued to deliver HPV vaccines at comparable rates 4 months after the initial intervention, even in the absence of additional training or interaction with the study team. Finally, the intervention was delivered at a very low cost, beyond the cost of the vaccine itself. Taken together, our findings suggest that this intervention is a promising approach to increasing Soldiers’ access to HPV vaccination and warrants wider-scale evaluation and dissemination.

Despite our success in increasing HPV vaccine uptake, additional research is needed to understand why many Soldiers declined HPV vaccination. In our initial intervention period, we found that older Soldiers, ages 21–26, were more likely to decline HPV vaccine. Although we asked Soldiers about the reasons for declination during the follow-up period, most Soldiers reported declining for an unspecified “other reason.” It is possible that declination may have been related to the COVID-19 pandemic and heightened awareness and concerns about vaccines in general. Alternatively, declination may be related to a lack of HPV vaccine requirement in the military, in contrast to requirements for many other routine vaccines. As such, Soldiers may perceive HPV vaccine as less valuable than other vaccines, which would be consistent with prior research that indicates that the absence of school entry requirements may make HPV vaccination seem less important to parents compared to adolescent vaccines that are required by schools.^18^ Regardless of the reasons for declining HPV vaccine, Soldiers may benefit from additional opportunities to receive recommendations for HPV vaccination and, if needed, to discuss its safety and effectiveness.^19–23^ For example, future interventions could engage providers and nursing staff to routinely recommend HPV vaccination during the Period Health Assessment, a health maintenance visit that Soldiers complete annually.

Our feasibility study identified several additional considerations that may inform future research and practice to improve HPV vaccine uptake among military populations. First, tracking vaccination status in the military's electronic health record can be challenging. Vaccination records can be dispersed across five or more differing electronic medical record databases, including Medical Protection System® (MEDPROS), Armed Forces Health Longitudinal Technology Application® (AHLTA), Essentris®, Composite Health Care System II®, and CarePoint Health®. The lack of a centralized vaccine registry for military personnel suggests the need for validation studies to determine the proportion of soldiers who are unvaccinated in the short term, as well as longer-term improvements in the EHR to promote vaccine documentation and reminders. At the time of this manuscript, a new EHR is being deployed throughout the Military Health System that should streamline efforts to evaluate vaccine status.^24^ Our study also highlights the importance of vaccination requirements, and future research can examine the feasibility of extending such requirements to HPV vaccination by evaluating the acceptability of such policies among military personnel.

This study is, to our knowledge, the first to evaluate the feasibility of a nursing-led intervention to increase HPV vaccine uptake among active-duty Soldiers, a population at particularly high risk for HPV infection. While these data may be used to inform future research and practice, our findings must be interpreted in light of several limitations. First, this single-site study focused on delivering HPV vaccinations during medical processing. Additional research will be needed to more broadly evaluate our intervention on other military bases, in different types of military clinics and in comparison to other types of interventions – for example, an education-only intervention in a site that already offers HPV vaccination may have a different effect. Second, the generalizability of our findings to nonmilitary contexts is unknown; such interventions could entail greater cost, given that the military purchases HPV vaccines at a discounted price and that vaccine doses contributed to the vast majority of intervention costs. Third, as a feasibility study, this project was limited in the scope of data collection. Larger samples of nurse participants would be needed to capture more subtle intervention effects. Similarly, more detailed demographic data, including race/ethnicity, will be important in future studies for characterizing our intervention’s promise for increasing equity in vaccination services. Finally, we conducted our study during the COVID-19 pandemic, which may have changed Soldiers’ and nursing staff members’ willingness to vaccinate. Future research will be needed to determine the uptake of HPV vaccine among soldiers after the pandemic and alongside COVID-19 vaccinations.

## Conclusion

Our findings suggest that training nursing staff to recommend and administer HPV vaccines is a highly feasible and low-cost way to sustainably increase Soldiers’ access to HPV vaccination. Until HPV vaccine is a required vaccine for military service, this intervention is a light-touch way to promote HPV vaccines among Soldiers. Given its promise, it warrants wider-scale testing as a strategy to increase military readiness and to protect Soldiers from HPV-attributable cancers.

## Supplementary Material

Supplemental Material

## Data Availability

The datasets generated during and/or analyzed during the current study are available from the corresponding author on reasonable request.

## Abbreviations

MEDPROS: Medical Protection System®
AHLTA: Armed Forces Health Longitudinal Technology Application®
HPV: human papillomavirus
MMR: Measles, Mumps and Rubella
EHR: electronic health record
SQUIRE: Standards for QUality ImprovementReporting Excellence
