# Supplementary figures and images for: Feasibility and sustainability of a nurse-led intervention to integrate HPV vaccination into medical processing for active-duty Soldiers
Source: Hum Vaccin Immunother. 2022 Dec 20;18(7):2153536. doi: 10.1080/21645515.2022.2153536 (PMC9891672; doi:10.1080/21645515.2022.2153536)

**Supplemental Figure 1**

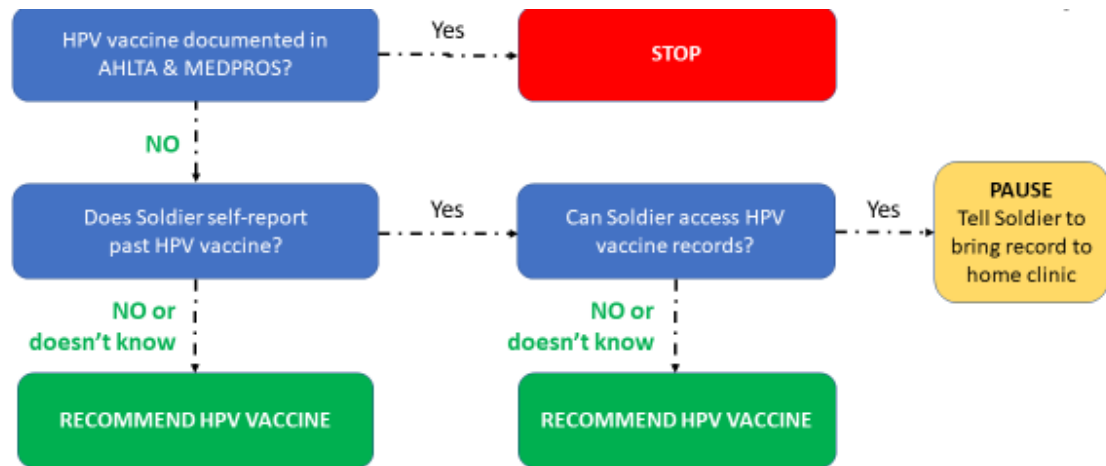

Supplement: Supplemental Material [file KHVI_A_2153536_SM7899.pdf]
